# Use of pediatric thymus to humanize mice for HIV-1 mucosal transmission

**DOI:** 10.1038/s41598-023-44366-2

**Published:** 2023-10-10

**Authors:** Chandra N. Roy, Sherry T. Shu, Christopher Kline, Lora Rigatti, Thomas E. Smithgall, Zandrea Ambrose

**Affiliations:** 1 Department of Microbiology and Molecular Genetics, University of Pittsburgh School of Medicine, Pittsburgh, PA USA; 2 Division of Laboratory Animal Resources, University of Pittsburgh School of Medicine, Pittsburgh, PA USA

**Keywords:** Microbiology, Virology, Retrovirus, Infectious diseases, HIV infections

## Abstract

Humanized mice have been used to study human immunodeficiency virus type 1 (HIV-1) transmission, pathogenesis, and treatment. The ability of pediatric thymus tissue implanted either in the leg (Leg PedThy) or under the renal capsule (Renal PedThy) with allogeneic CD34+ hematopoietic cells (HSCs) in NSG mice was evaluated for reconstitution of human immune cells and for rectal transmission of HIV-1. These mice were compared to traditional BLT mice implanted with fetal liver and thymus under the renal capsule and mice injected only with HSCs. Renal PedThy mice had similar immune reconstitution in the blood, spleen and intestine as BLT mice, while Leg PedThy mice had transient detection of immune cells, particularly CD4+ T cells and macrophages, the target cells for HIV-1 infection. Rectal transmission and replication of HIV-1 was efficient in BLT mice but lower and more variable in Renal PedThy mice. HIV-1 was poorly transmitted in HSC mice and not transmitted in Leg PedThy mice, which correlated with the frequencies of target cells in the spleen and intestine. Humanization of NSG mice with pediatric thymus was successful when implanted under the kidney capsule, but led to less efficient HIV-1 rectal transmission and replication compared to BLT mice.

## Introduction

Globally there are still more than 39 million people infected with human immunodeficiency virus type 1 (HIV-1)^1^, which has no reliable cure. Even with effective antiretroviral therapy (ART), 25% of HIV-infected people do not receive it, which led to 1.3 million new infections and 630,000 HIV-related deaths in 2022^1^. Thus, there is still a need for better prevention and treatment methods until an effective cure is discovered.

Animal models are critical for studying human disease mechanisms, prevention, and treatment. However, HIV-1 does not efficiently infect non-human cells due to numerous species-specific host factor differences^2,3^. Mice with humanized immune systems (HIS mice) allow the study of HIV-1 transmission, prevention methods, pathogenesis, co-morbidities, and therapies in the context of an immune system and intact tissues. HIS models to study HIV-1 in vivo include human CD34+ hematopoietic stem cell (HSC) transplantation into immunodeficient mice, such as NOD.Cg-*Prkdc*^*scid*^*Il2rg*^*tm1Wjl*^/SzJ (NSG) or NOD.Cg-*Prkdc*^*scid*^*Il2rg*^*tm1Sug*^/JicTac (NOG) mice^4–6^. HSCs differentiate in vivo into B and T lymphocytes and, to a lesser extent, myeloid cells. Generally, reconstitution of T cells is most efficient when HSCs are derived from fetal liver or cord blood rather than from granulocyte–macrophage colony-stimulating factor (GM-CSF)-mobilized adult blood or bone marrow-derived HSCs from adults^7^. However, myeloid cell frequency as well as T lymphocyte functionality and reconstitution of immune cells in mucosal tissues is dramatically improved by transplantation of HSCs with either implantation of fetal liver and thymus tissues under the renal capsule (BLT mice)^4^ or after genetic modification of the mice to produce one or more human proteins *in vivo*^5^.

As access to human fetal tissues for research can be challenging or even impossible in some locations, neonatal or pediatric thymus removed during cardiac surgery has been recently assessed as an alternative to fetal thymus to produce HIS mice. To study induced pluripotent stem cell therapy, Brown et al. produced a NeoThy mouse model with HSC injection with autologous or allogeneic thymus tissue from neonates (median age of 7 days), which were implanted under the kidney capsule of irradiated NSG mice or non-irradiated NOD.Cg-*Prkdc*^*scid*^*Il2rg*^*tm1Wjl*^/SzJ*-Kit*^*W41/W41*^ (NSG-W) mice^8^. Immune reconstitution of CD45+ cells and differentiation into CD3+ and CD19+ cells was similar in the blood of mice implanted with fetal or neonatal thymus. Lifespans of mice implanted with allogeneic HSCs and thymus samples were improved with injection of anti-CD2 antibody to prevent graft vs. host disease (GVHD). However, HIV-1 transmission and replication was not assessed in this model. Colas et al. created the Cord blood and Cardiac Surgery Thymus (CCST) model in which pediatric thymus was cultured for 7–10 days to remove thymocytes and implanted in the quadriceps muscle prior to injection of allogeneic HSCs^9^. Implantation in the leg muscle is technically less challenging compared to implantation under the renal capsule, which makes this variation attractive. While immune reconstitution and functionality of CD4+ and CD8+ T cells in the blood and tissues was greater in BLT mice compared to CCST mice, systemic and mucosal HIV-1 challenge of mice led to similar infection rates and viral replication^9^.

Here we compare immune reconstitution and rectal transmission of HIV-1 in NSG mice implanted with pediatric thymus in the quadriceps muscle (Leg PedThy) or under the renal capsule (Renal PedThy) with allogeneic HSCs. These animals were compared to mice injected with HSCs without thymus implantation (HSC mice) and traditional BLT mice. We observed only transient immune reconstitution in Leg PedThy mice, whereas many Renal PedThy mice had similar but somewhat lower immune reconstitution compared to BLT mice. Rectal transmission and replication of HIV-1 in all animals correlated with reconstitution of intestinal CD68+ macrophages and CD4+ T cells in the intestine and spleen.

## Results

### Immune reconstitution of PedThy mice was improved with renal implantation compared to implantation in the leg

**Figure 1:**
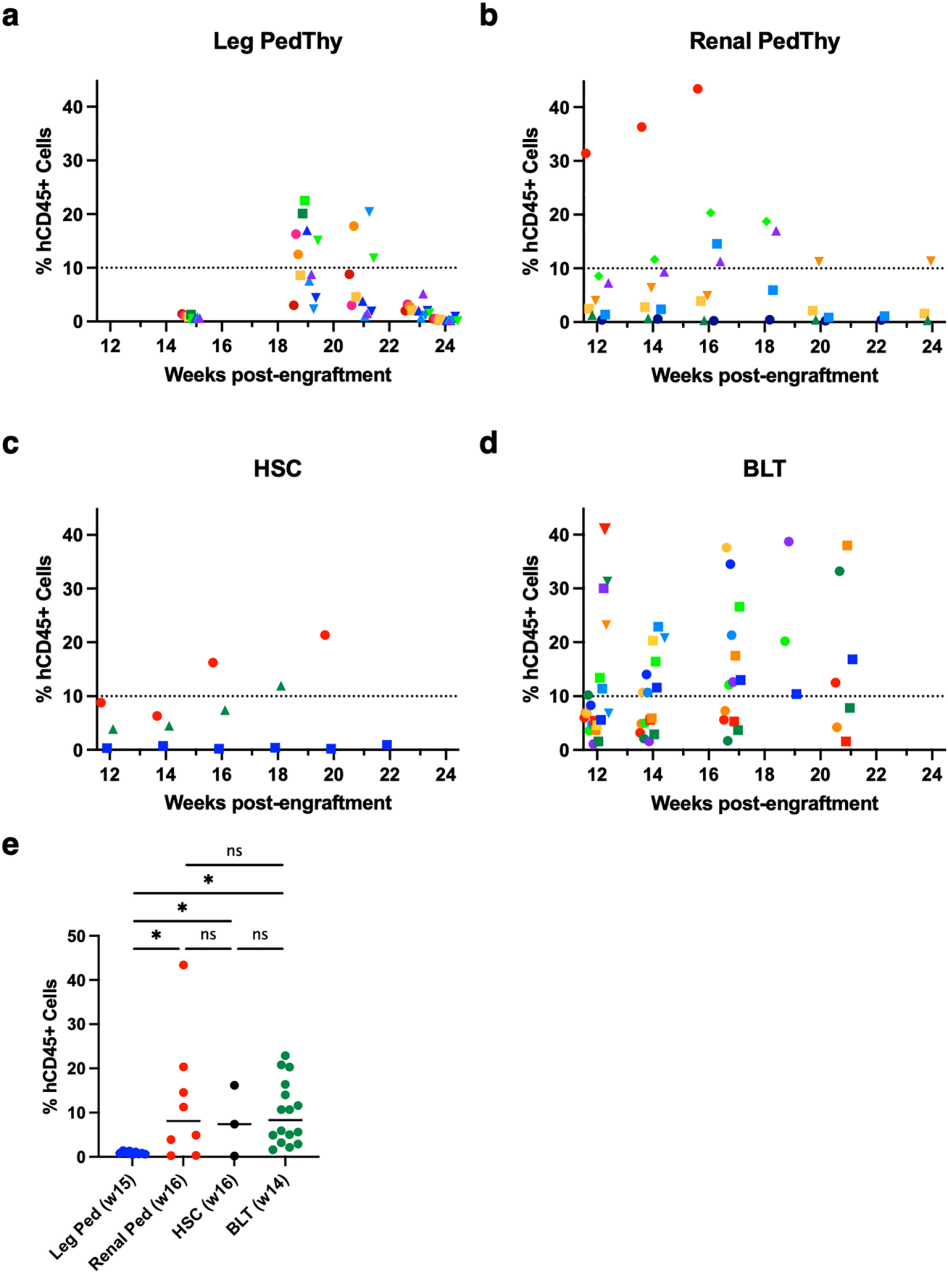
Human CD45+ cells detected in the peripheral blood of mice. (**A**–**D**) The percentage of hCD45+ cells in the peripheral blood are shown over time for (**A**) Leg PedThy produced with irradiation (●), with busulfan (■), no myeloablation (▲) or with busulfan and anti-CD2 antibodies (▼); (**B**) Renal PedThy; (**C**) HSC; and (**D**) BLT mice described in Table 1. Once reconstitution was reached, animals were challenged with HIV-1 and flow cytometry was no longer performed. (**E**) The percentage of hCD45+ cells in the peripheral blood at week 14–16 for each animal is shown and compared. * denotes *p* values ≤ 0.05; ns denotes *p* > 0.05. Each symbol denotes a separate animal.

Leg PedThy mice were produced by surgical engraftment of human pediatric thymus from a single donor (Donor 1) into the quadriceps muscle followed by HSC injection (Table 1), as previously described^9^. To compare the effects of myeloablation on immune reconstitution, mice were myeloablated with sublethal irradiation (n = 3) or busulfan injection (n = 6) or were not myeloablated (n = 3) and followed for up to 24 weeks post-engraftment. To ensure that potential remaining thymocytes in the engrafted tissue were not causing transient immune reconstitution, half of the busulfan-treated mice were injected with anti-CD2 antibodies prior to thymus engraftment (n = 3) (Table 1 and Fig. 1A), as described previously^8^. At necropsy, no discernable thymic tissue was grossly observed at the surgical site of any of the animals. Mice were considered reconstituted when they had greater than 10% human (h) CD45+ cells in the blood determined by flow cytometry (Supplementary Fig. S1), as previously described^10^. Frequencies of human CD45+ cells were below 1.5% at week 15 post-engraftment (0.5–1.4%; Fig. 1A). At week 19, hCD45+ cells had increased substantially in all animals (3.0–22.5%) with 7/12 animals having > 10% human peripheral blood cells. One or two animals from each myeloablation group reached the reconstitution threshold. However, the frequency of hCD45+ cells decreased to near undetectable levels in all animals by week 24 (0.2–0.5%). Myeloablation did not appear to make a difference in immune reconstitution, with animals either remaining unreconstituted or becoming only transiently reconstituted.

**Table 1:** Animal information.

Animal	Thymus implantation	Thymus source^a^	Sex	Myeloablation	Immune reconstitution^b^	HIV-1 infected
CC09	Leg	Pediatric, Donor 1	F	Irradiation	No (9%)	nd
CC10		Pediatric, Donor 1	F	Irradiation	Transient (16%)	nd
CC11		Pediatric, Donor 1	F	Irradiation	Transient (18%)	nd
CC12		Pediatric, Donor 1	M	Busulfan	No (9%)	nd
CC13		Pediatric, Donor 1	M	Busulfan	Unknown (20%)	No^c^
CC14		Pediatric, Donor 1	M	Busulfan	Unknown (23%)	No^c^
CC15		Pediatric, Donor 1	F	None	Transient (17%)	nd
CC16		Pediatric, Donor 1	F	None	No (8%)	nd
CC17		Pediatric, Donor 1	F	None	No (9%)	nd
CC19^d^		Pediatric, Donor 1	M	Busulfan	Transient (20%)	nd
CC20^d^		Pediatric, Donor 1	M	Busulfan	No (4%)	nd
CC21^d^		Pediatric, Donor 1	M	Busulfan	Transient (15%)	nd
CC22	Renal capsule	Pediatric, Donor 1	F	Busulfan	Yes (43%)	Yes
CC23		Pediatric, Donor 1	F	Busulfan	No (4%)	nd
CC24		Pediatric, Donor 1	F	Busulfan	No (1%)	nd
CC25		Pediatric, Donor 1	F	Busulfan	Yes (11%)	Yes
CC29		Pediatric, Donor 2	F	Busulfan	Yes (19%)	Yes
CC30		Pediatric, Donor 2	F	Busulfan	No (0.4%)	nd
CC31		Pediatric, Donor 2	F	Busulfan	Transient (15%)	nd
CC32		Pediatric, Donor 2	F	Busulfan	Yes (17%)	Yes
CC27	None	-	F	Busulfan	Yes (21%)	Yes
CC33		-	F	Busulfan	No (0.9%)	nd
CC34		-	M	Busulfan	Yes (12%)	Yes
152	Renal capsule	HFT, Donor A	F	Irradiation	Yes (13%)	nd
153		HFT, Donor A	F	Irradiation	No (7%)	nd
154		HFT, Donor A	F	Irradiation	Yes (38%)	nd
156		HFT, Donor A	F	Irradiation	Yes (33%)	nd
157		HFT, Donor A	F	Irradiation	Yes (20%)	nd
158		HFT, Donor A	F	Irradiation	Yes (35%)	nd
159		HFT, Donor A	F	Irradiation	Yes (21%)	nd
160		HFT, Donor A	F	Irradiation	Yes (39%)	nd
161		HFT, Donor A	F	Irradiation	No (5%)	nd
162		HFT, Donor A	F	Irradiation	Yes (38%)	nd
163		HFT, Donor A	F	Irradiation	Yes (20%)	nd
164		HFT, Donor A	F	Irradiation	No (8%)	nd
165		HFT, Donor A	F	Irradiation	Yes (27%)	nd
166		HFT, Donor A	F	Irradiation	Yes (13%)	nd
316		HFT, Donor B	F	Irradiation	Yes ( 30%)	Yes
317		HFT, Donor B	F	Irradiation	Yes ( 23%)	Yes
318		HFT, Donor B	F	Irradiation	Yes ( 23%)	Yes
319		HFT, Donor B	F	Irradiation	Yes ( 21%)	Yes
320		HFT, Donor B	F	Irradiation	Yes ( 20%)	Yes
322		HFT, Donor B	F	Irradiation	Yes ( 21%)	Yes

*HFT* human fetal tissue.

*nd* not done (intrarectal challenge not performed).

^a^Ages of Donors 1 and 2 were 3 years and 11 weeks, respectively.

^b^Reconstitution defined as peripheral hCD45+ cell percentage > 10% maintained for more than 1 time point (highest percentage of peripheral hCD45+ cells indicated).

^c^Mice had undetectable plasma viremia after two HIV-1 rectal challenges.

^d^Anti-CD2 antibody administered.

For comparison, Renal PedThy mice were produced with thymus from the same donor (Donor 1; n = 4) used to produce the Leg PedThy animals or from a thymus from a younger donor (Donor 2; n = 4; Table 1). The animals were myeloablated with busulfan prior to thymus engraftment under the kidney capsule. At necropsy, a 4–6 mm diameter white-tan focus was observed at the surgical site on each of the kidneys of the Renal PedThy mice, although it was smaller than those seen in BLT mice (Supplementary Fig. S2A). Hematoxylin and eosin (H&E) staining of representative BLT kidneys showed various sizes of remaining lymphoid tissue consistent with the thymic graft (Supplementary Fig. S2B, top). Surprisingly, only one of four Renal PedThy kidneys (CC25) showed histologic evidence of functional subcapsular lymphoid tissue, while the other kidneys did not have discernible remaining lymphoid tissue (Supplementary Fig. S2B, bottom). The kidney from animal CC22 had a focus of white adipose tissue that extended into the parenchyma, while CC29 had thickening of fibrous connective tissue at the surgical site on the renal capsule. Interestingly, CC32 had a subcapsular focus of non-lymphoid cells surrounded by irregular fibrous connective tissue. These non-lymphoid cells have a histomorphology consistent with epithelial cells, potentially thymic epithelial remnant tissue, or histiocytic inflammatory cells.

In contrast to the Leg PedThy animals, 2/8 animals had > 10% hCD45+ cells by week 14 post-engraftment (Fig. 1B). Ultimately 4/8 animals became reconstituted with increasing or plateauing hCD45+ cell frequencies and another animal having transient reconstitution with its hCD45+ cells decreasing to basal levels by week 20. Similarly, 2/3 HSC (Fig. 1C) and several BLT (Fig. 1D) mice had similar immune reconstitution. No significant differences in hCD45+ frequencies between the Renal PedThy, HSC, and BLT animals at week 14 -16 post-engraftment were seen (Fig. 1E). In contrast, the Leg PedThy animals had no detectable immune reconstitution at week 15, which was significantly delayed compared to the other groups and was ultimately transient.

### Renal PedThy mice had better peripheral T and B lymphocyte reconstitution than Leg PedThy mice

**Figure 2:**
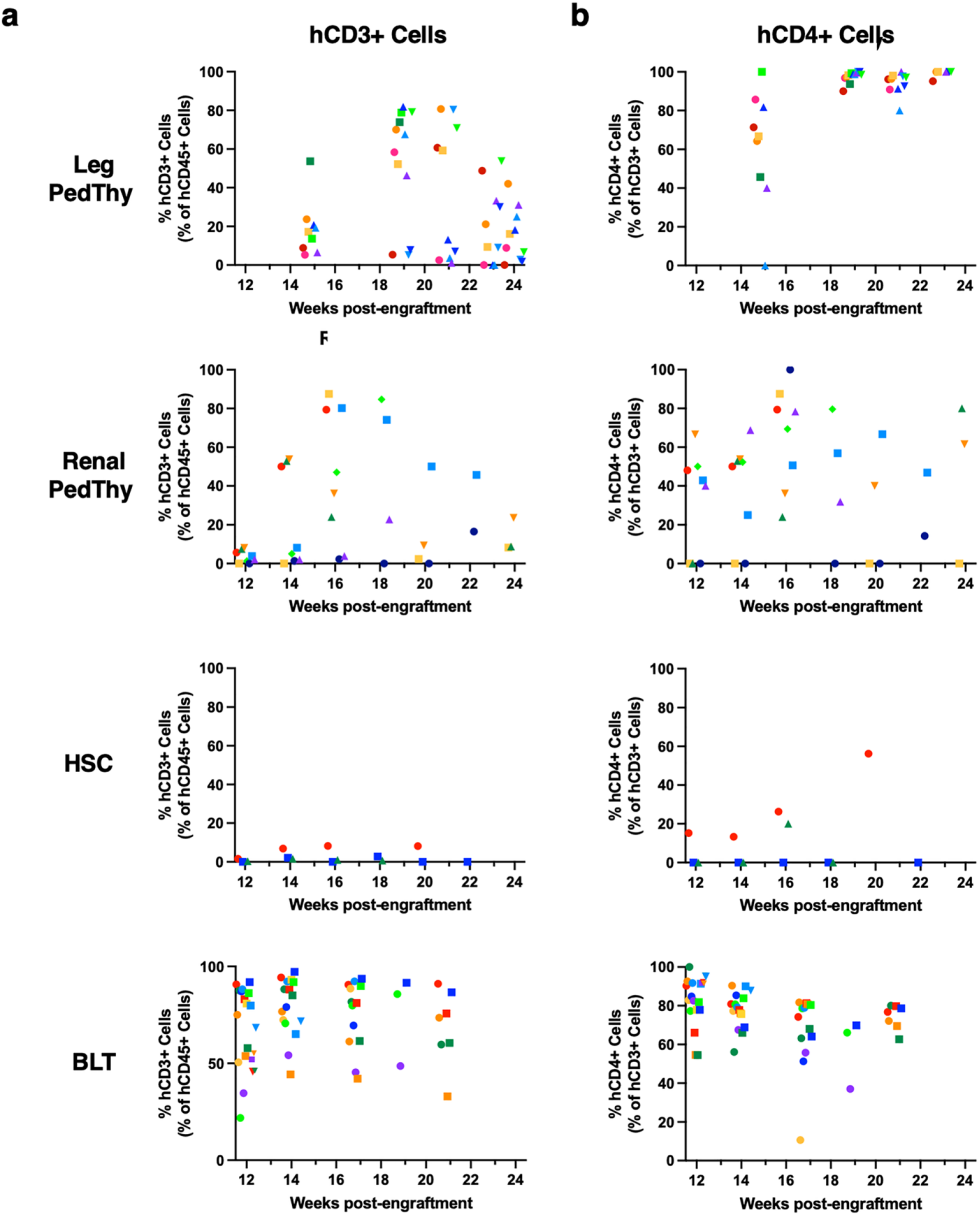
Human CD3+CD4+ cells detected in the peripheral blood of mice. The percentage of (**A**) hCD3+ cells in the peripheral hCD45+ cell population and (**B**) hCD4+ cells in the hCD3+ cell population are shown over time for the Leg PedThy, Renal PedThy, HSC, and BLT mice described in Table 1. Each symbol denotes a separate animal. Once reconstitution was reached, animals were challenged with HIV-1 and flow cytometry was no longer performed.

Human lymphocyte subsets were measured over time in all of the mice to understand the quality of immune reconstitution. Overall peripheral T lymphocyte (CD3+) frequencies and kinetics were similar in the Leg and Renal PedThy animals, which were lower than those observed in BLT mice and higher than in HSC mice (Fig. 2A). As CD4+ T cells are targets of HIV-1 infection, these cells are important in a model to study infection or transmission. Whereas CD4+ T cells increased to 93.7–100% of all T cells in the Leg PedThy mice, lower and more variable CD4+ T cell frequencies were observed in the Renal PedThy, HSC, and BLT groups (Fig. 2B), which suggest that the frequencies of other T cell subsets were much lower in Leg PedThy model than the other models.

As expected, CD3+CD8+ T cell frequencies were low or undetectable at all but a single time point in two animals in the Leg PedThy group (Supplemental Fig. S3A). In contrast, CD3+CD8+ T cells were observed in most PedThy Renal, HSC, and BLT mice (Supplemental Fig. S3A,B,C, respectively). Overall, Renal PedThy mice had a mixture of CD4+ and CD8+ T cells over time similar to BLT and HSC mice, while Leg PedThy mice had mostly CD4+ T cells.

**Figure 3:**
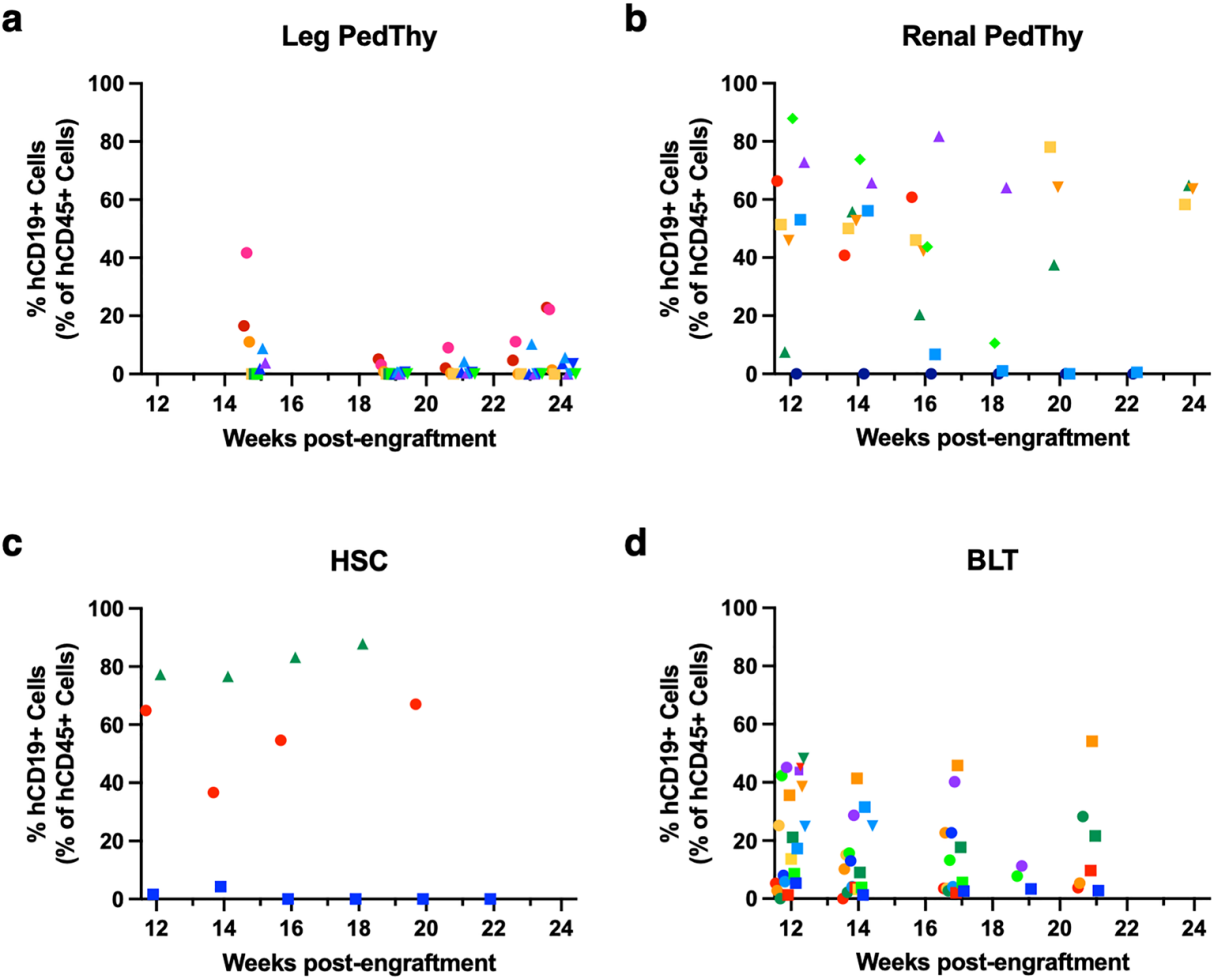
Human CD19+ cells detected in the peripheral blood of mice. The percentage of hCD19+ cells in the peripheral hCD45+ cell population are shown over time for (**A**) Leg PedThy, (**B**) Renal PedThy, (**C**) HSC, and (**D**) BLT mice described in Table 1. Each symbol denotes a separate animal. Once reconstitution was reached, animals were challenged with HIV-1 and flow cytometry was no longer performed.

Human B lymphocytes were measured by CD19 staining. Leg PedThy mice had low frequencies of B cells (< 42%) that remained low or declined over time (Fig. 3A). In contrast, 7/8 Renal PedThy mice had much higher B cell frequencies (7.5–87.9%) that remained high in all but one animal (Fig. 3B). One animal had no detectable B cells over time. Renal PedThy mice had similar frequencies to those seen in HSC (Fig. 3C) and BLT (Fig. 3D) mice, suggesting that the Leg PedThy mice had inferior B lymphocyte development compared to the other models tested.

### CD3-CD19- cell frequency is inversely correlated with human CD45+ cell reconstitution

**Figure 4:**
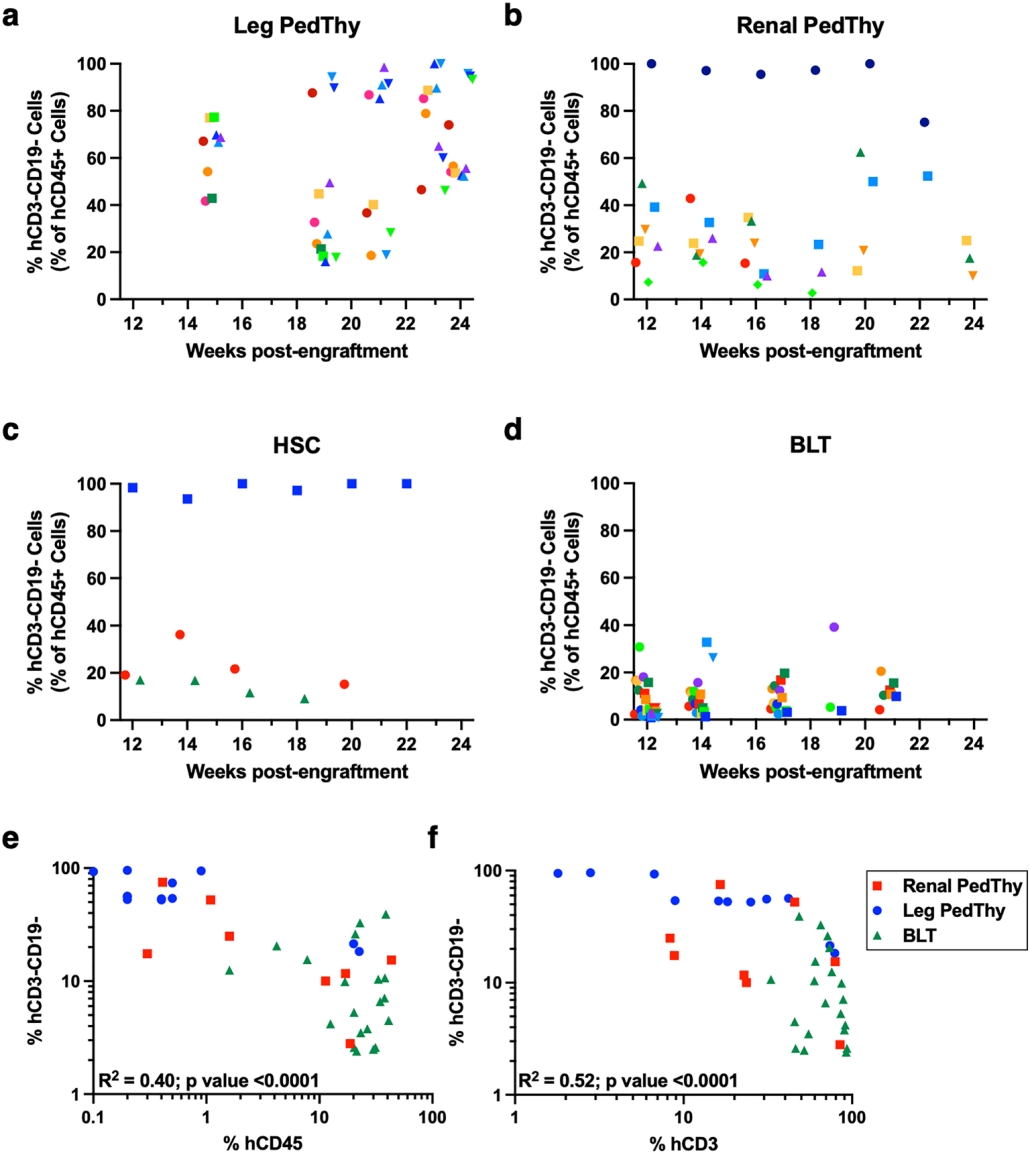
Human CD3-CD19- cells detected in the peripheral blood of mice. (**A**–**D**) The percentage of hCD3-CD19- cells in the peripheral hCD45+ cell population are shown over time for (**A**) Leg PedThy, (**B**) Renal PedThy, (**C**) HSC, and (**D**) BLT mice described in Table 1. (**E**–**F**) The correlation of the percentages of hCD3-CD19- cells with (E) hCD45+ cells or (F) hCD3+ cells are shown for the last tested time point for the Leg PedThy, Renal PedThy, and BLT mice. Pearson correlation coefficients and 2-tailed *p* values are shown. Each symbol denotes a separate animal. Once reconstitution was reached, animals were challenged with HIV-1 and flow cytometry was no longer performed.

Besides CD3+ T and CD19+ B cells, the lymphocyte gate contained hCD3-CD19- cells. These cells are likely triple negative (CD3-CD4-CD8-) T cell progenitors^11^ and perhaps low frequencies of defective NK cells^12,13^. In Leg PedThy mice, the proportion of hCD45+ cells that were negative for both CD3 and CD19 ranged from 41.7 to 77.3% at week 15 post-engraftment (Fig. 4A). Overall, the frequency of this cell population remained high in this group over time. In contrast, these cells were more variable in Renal PedThy mice with 7/8 having frequencies of 15.7–42.9% and one animal having 97.1% at week 14 (Fig. 4B). This population either decreased or remained stable in all Renal PedThy animals over time. This was similar in the HSC (Fig. 4C) and the BLT (Fig. 4D) groups.

It appeared that mice with the poorest immune reconstitution, regardless of humanization method, had the highest frequencies of CD3-CD19- cells. To determine if this cell population was associated with lower immune reconstitution, we plotted the frequencies of CD3-CD19- cells against the frequencies of hCD45+ (Fig. 4E) or CD3+ (Fig. 4F) cells for each mouse in all groups at the last time point tested. Indeed, CD3-CD19- cell frequencies were inversely correlated with hCD45+ and CD3+ cell frequencies, suggesting that these triple negative cells represent progenitor T cells that do not differentiate in the mice.

### Monocyte/macrophage cell frequencies in Renal PedThy and BLT mice were similar and higher than in the other groups

**Figure 5:**
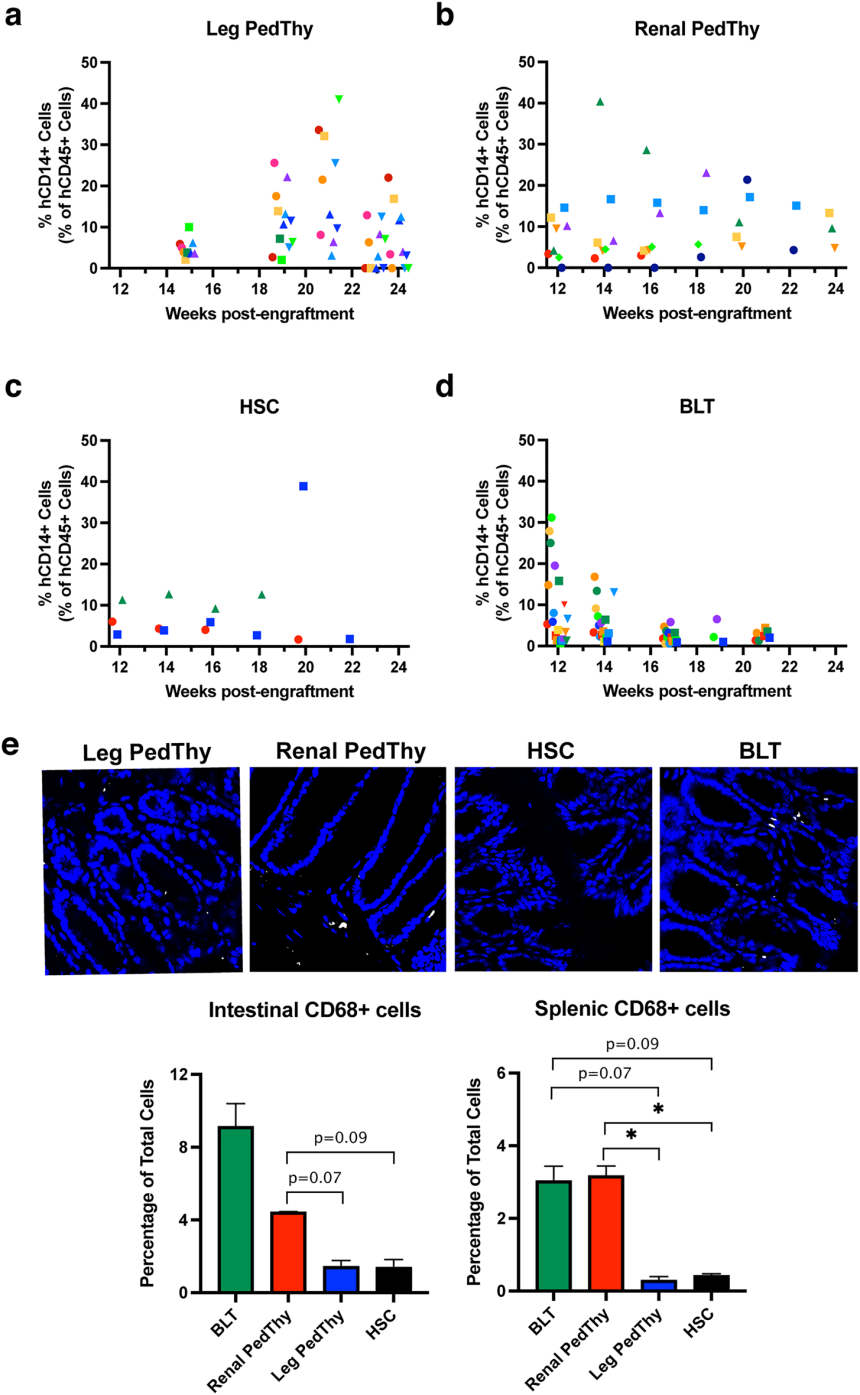
Human CD14+ cells detected in the peripheral blood of mice. The percentage of hCD14+ cells in the peripheral hCD45+ cell population are shown over time for (**A**) Leg PedThy, (**B**) Renal PedThy, (**C**) HSC, and (**D**) BLT mice described in Table 1. Each symbol denotes a separate animal. Once reconstitution was reached, animals were challenged with HIV-1 and flow cytometry was no longer performed. (**E**) Immunofluorescence staining of CD68+ cells (white) and nuclear DAPI stain (blue) in representative sections of intestine from a mouse from each group are shown. The average percentages of CD68+ cells per field of total cells are shown from spleen and intestine sections of mice from each group (n = 2, 5 fields of view/tissue). * denotes *p* values ≤ 0.05; *p* values of 0.06–0.09 are shown.

Myeloid cell reconstitution of HIS mice is generally improved in the BLT model compared to mice injected with only HSCs^4,5^. Peripheral human CD14+ monocytes were measured in the four groups of mice. The frequencies of monocytes in Leg PedThy mice increased from 2-10% at week 15 to 3–41% by week 21, but plateaued or decreased thereafter (Fig. 5A). Renal PedThy mice had hCD14+ cell frequencies of 0–40% at week 14, which was maintained or decreased to 5–21% at week 20 (Fig. 5B). In contrast, monocytes generally remained low (2–13%) between 12 to 22 weeks in HSC mice with the exception of one time point in one animal (Fig. 5C). BLT mice had monocyte frequencies of 1–31% at week 12 that decreased in all animals over time (Fig. 5D), which could reflect migration into tissues to form macrophages.

Human CD68+ macrophages were stained and visualized in spleen and intestine at necropsy by immunofluorescence microscopy in representative mice from each group (Fig. 5E). Leg PedThy mice had low macrophage frequencies in the spleen, which were similar in HSC mice. In contrast, Renal PedThy mice had similar frequencies of macrophages to BLT mice, which were significantly different than those observed in the Leg PedThy mice. In the intestine, Leg PedThy and HSC mice had lower macrophage frequencies than Renal PedThy mice and BLT mice. Overall, these results suggest that human macrophage reconstitution was greater in Renal PedThy and BLT mice compared to Leg PedThy and HSC mice.

### Rectal HIV-1 transmission requires stable human cell engraftment

**Figure 6:**
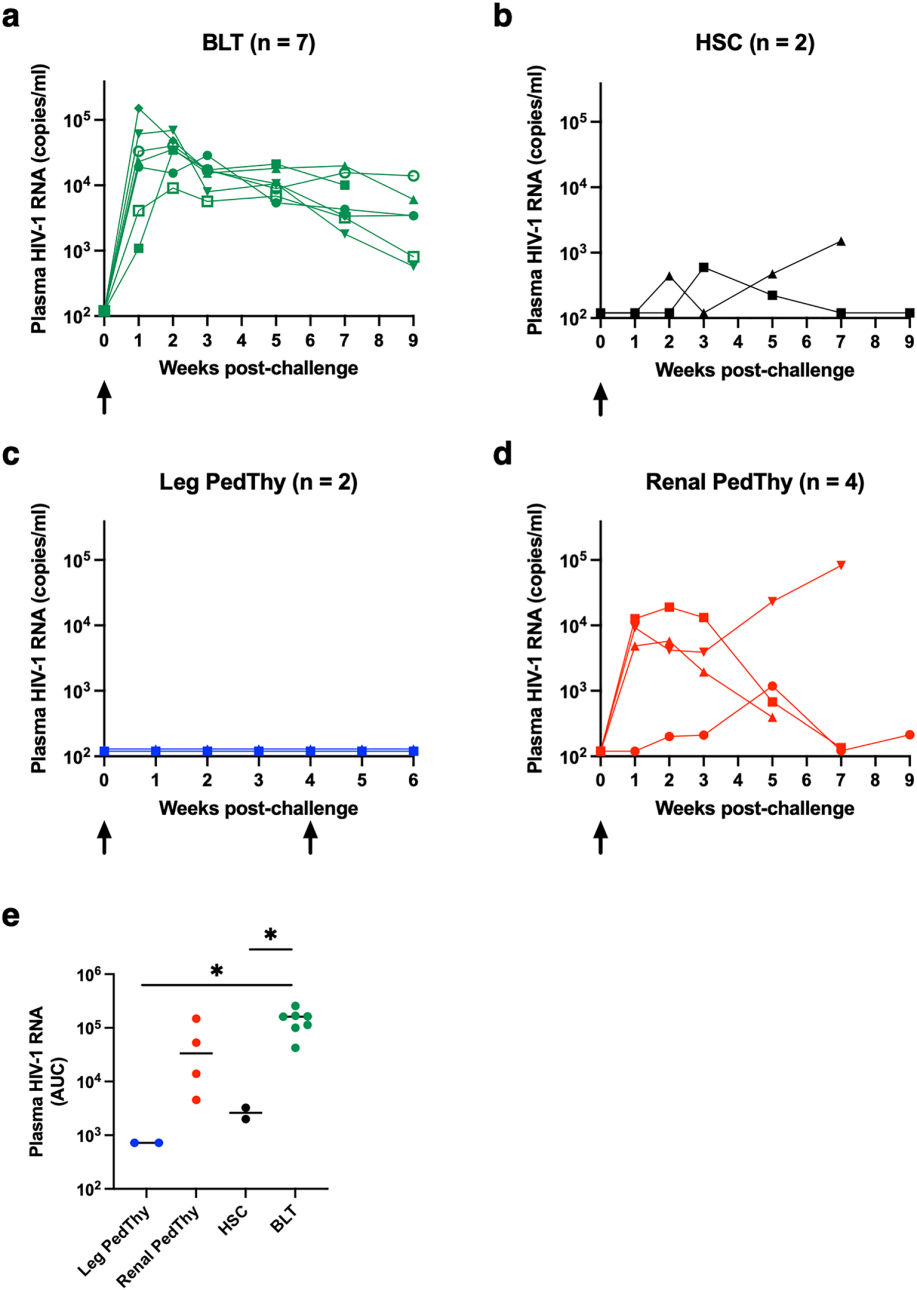
Plasma viremia of mice challenged intrarectally with HIV-1. (**A**–**D**) Plasma HIV-1 RNA copies are shown over time for (**A**) BLT, (**B**) HSC, (**C**) Leg PedThy, and (**D**) Renal PedThy mice that were challenged with HIV-1. Each line depicts a different mouse. (**E**) The plasma viremia area under the curve (AUC) is shown for each mouse. Each symbol denotes a separate animal. * denotes *p* values ≤ 0.05.

As the vast majority of new HIV-1 infections in the United States occur in men due to male-to-male sexual contact^14^, we evaluated rectal transmission of a subtype C transmitted founder (T/F) HIV-1 strain, HIV-1_CH185_, that we previously used for vaginal transmission in BLT mice^15^. Rectal challenge of seven BLT mice led to all animals becoming infected with peak plasma viremia levels of 9.1 × 10^3^–1.5 × 10^5^ RNA copies/ml at 1–3 weeks post-challenge (Fig. 6A). Similar challenge of HSC mice led to low, intermittent detectable plasma viremia (6.0 × 10^2^–1.5 × 10^3^ RNA copies/ml; Fig. 6B), which is consistent with fewer CD4+ target cells in the mucosa in the absence of fetal thymus implantation. Two Leg PedThy mice that became reconstituted with human CD45+ cells (CC13 and CC14) were challenged intrarectally but no plasma viremia was detected by 3 weeks (Fig. 6C). A second rectal challenge of these animals was performed and the mice still remained uninfected. To ensure that reconstitution of the Leg PedThy mice was not transient, additional mice in this group were evaluated for reconstitution for an additional time point prior to HIV-1 challenge. However, the rest of the Leg PedThy mice did not maintain hCD45+ cells above 10% frequency and, thus, were not challenged. In contrast, Renal PedThy mice that had > 10% hCD45+ cell frequencies at two time points (n = 4) were also rectally challenged with HIV-1 and all became infected (Fig. 6D). Three of the Renal PedThy mice had peak plasma viremia levels of 5.8 × 10^3^–1.9 × 10^5^ RNA copies/ml at 1–2 weeks post-challenge, while the fourth had low viremia similar to the HSC mice, despite having the highest hCD45+ cell reconstitution in the Renal PedThy group. BLT mice had similar areas under the curve (AUC) for plasma viremia, the mean of which was the highest of all groups (1.4 × 10^5^) and significantly different than those of the Leg PedThy and HSC groups (7.2 × 10^2^ and 2.6 × 10^3^, respectively; Fig. 6E). Renal PedThy AUC levels were more variable, the mean of which was 5.5 × 10^4^.

**Figure 7:**
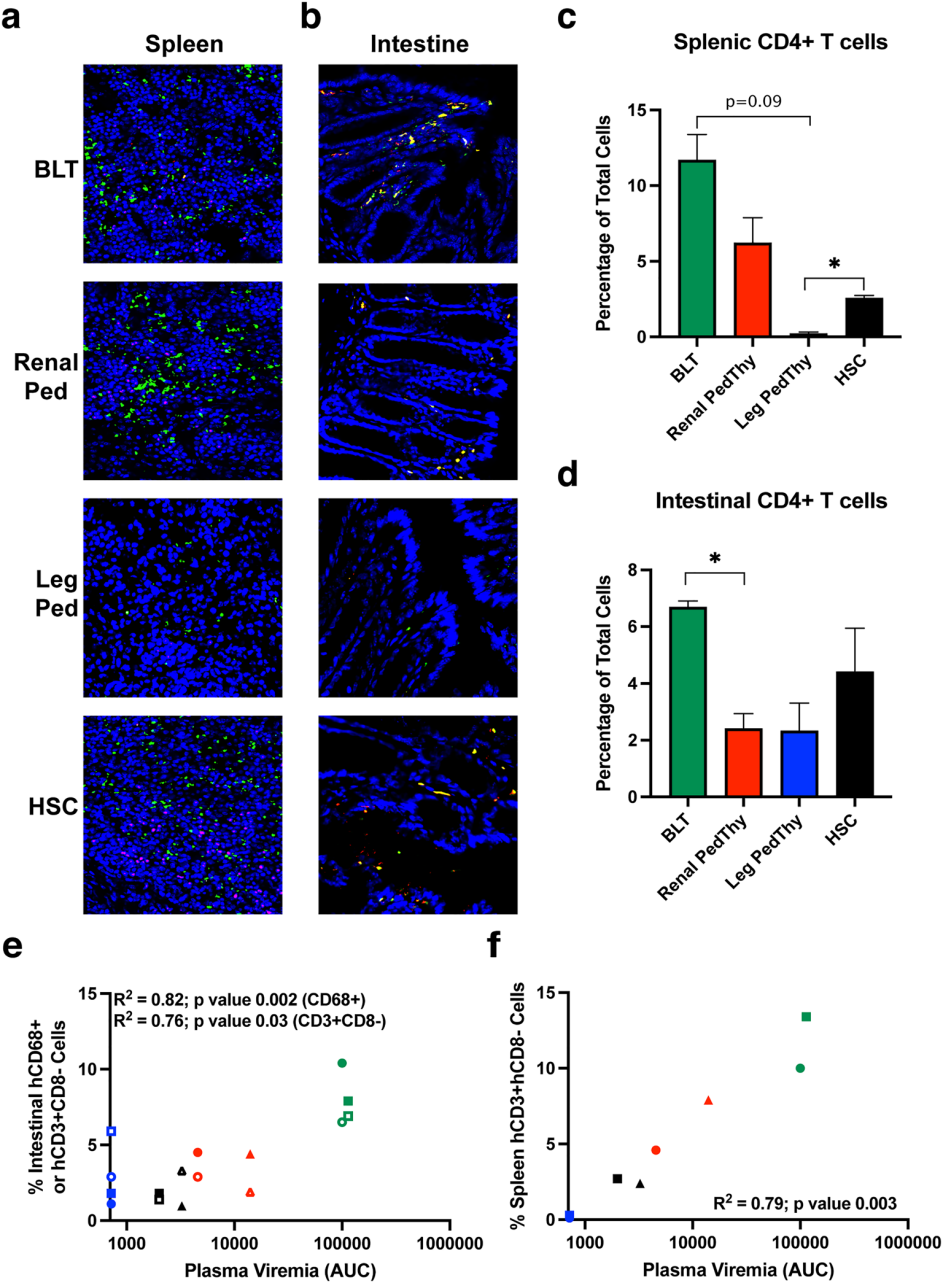
Human CD4+ and CD8+ T cells detected in spleen and intestine of HIV-challenged mice. (**A**–**B**) Immunofluorescence staining of hCD3+ (red) and hCD8+ (green) cells in (**A**) spleen and (**B**) intestine sections from representative mice in each group is shown. (**C**–**D**) The average percentages of (**C**) splenic and (**D**) intestinal CD3+CD8- cells per field of total cells are shown from mice from each group (n = 2, 5 fields of view/tissue). * denotes *p* values ≤ 0.05; *p* values of 0.06–0.09 are shown. (**E**–**F**) The correlations of plasma viremia AUC with the frequencies of (**E**) intestinal hCD68+ cells (solid symbols) and hCD3+CD8- cells (open symbols) or (**F**) splenic hCD3+CD8- cells are shown. Each symbol represents a different animal in the BLT (green), Renal PedThy (red), Leg PedThy (blue), and HSC (black) groups. Pearson correlation coefficients and 2-tailed *p* values are shown.

As the main target cells for HIV-1 are human CD4+ T cells, we estimated the frequencies of those cells by immunofluorescence in the spleens (Fig. 7A) and intestines (Fig. 7B) of mice challenged with virus after necropsy. Because HIV-1 downregulates CD4 expression in cells, hCD3+CD8- cells were used as an indirect measure of hCD4+ T cells. BLT mice had the highest frequencies of hCD4+ T cells in the spleen that were not significantly different than the frequencies observed in Renal PedThy mice (Fig. 7C). Leg PedThy mice had very few splenic hCD4+ T cells, which was not surprising given the transient T cell levels detected in peripheral blood (Fig. 2A). HSC mice had significantly more splenic hCD4+ T cells than Leg PedThy mice, but less than the other groups. Relatively low frequencies of hCD4+ T cells were detected in the intestines of all mice (Fig. 7D) compared to hCD8+ T cells (Supplemental Fig. S4), which is consistent with intestinal intraepithelial lymphocytes^16^.

Previous studies concluded that the ability of BLT mice to become infected with HIV-1 after vaginal challenge corresponded to frequencies of hCD4+ T cells in the vaginal mucosa^10,17^. To determine the correlation of HIV-1 target cells with HIV-1 transmission and replication in the different mice in this study, the plasma viremia AUC was compared to frequencies of either hCD68+ cells or hCD4+ cells in the intestine, where infection would initiate, and spleen, the major lymphoid organ in which HIV-1 would replicate. Intestinal human macrophage frequencies were generally higher than hCD4+ T cells in the mice, but both were significantly correlated with plasma viremia (Fig. 7E). The level of viremia also correlated with frequencies of hCD4+ T cells in the spleen (Fig. 7F) but not with hCD68+ cells (data not shown). These results suggest that HIV-1 rectal transmission and subsequent dissemination rely on stable frequencies of human target cells in tissues, which were greatest in BLT mice followed by Renal PedThy mice.

## Discussion

The use of animal models for HIV-1 research has led to better understanding of viral pathogenesis and development of new drugs for PrEP and ART^18,19^. HIS mice are more tractable and economical than nonhuman primates, but they have limitations in terms of immune reconstitution, particularly for myeloid cells and at mucosal sites^5^. While newer transgenic mouse strains express human immune factors and do not require fetal tissue implantation for immune reconstitution^5,6^, the gold standard for HIV-1 studies in HIS mice has been BLT mice, which are produced by implantation of human fetal tissues and for improved engraftment in the blood and multiple tissues and immune functionality^5^. But the use of fetal tissues have sparked ethical concerns and, as a consequence, are not accessible to all researchers. In addition, donor variability, source of HSCs, myeloablation method, and the mouse strain may affect the reconstitution of human immune cells and their function.

Two recent studies have investigated the implantation of neonatal or pediatric thymus instead of fetal thymus to produce HIS NSG mice, with one implanting the thymus under the renal capsule in the same manner as BLT mice^8^ and the other implanting the tissue in the quadriceps muscle^9^, as is performed in infants with DiGeorge syndrome^20^. We sought to compare the two implantation methods in NSG mice with allogeneic HSCs and also compare with BLT and HSC mice. No significant differences in hCD45+ frequencies between the Renal PedThy, HSC, and BLT animals were observed at week 14–16 post-engraftment. However, the Leg PedThy animals had delayed immune reconstitution after week 15, which was ultimately transient compared to the other groups. Over all, BLT and Renal PedThy mice had better peripheral T lymphocyte reconstitution, particularly CD4+ T cells that are infected by HIV-1, than Leg PedThy mice, as measured in the blood by flow cytometry at multiple time points and in the spleen by immunofluorescence at necropsy. Ultimately, hCD3-CD19- cell frequencies, which likely represent undifferentiated progenitor T cells, were inversely correlated with hCD45+ and CD3+ T cell frequencies.

As myeloid cell development is significantly improved in BLT mice compared to HSC mice^21,22^ and macrophages represent another HIV-1 target cell, we compared the frequencies of CD14+ monocytes in the blood and CD68+ macrophages in tissues. While BLT and Renal PedThy mice had similar frequencies of monocytes in the blood at 12–14 weeks post-engraftment, they declined over time in BLT mice and remained relatively stable in Renal PedThy mice. While both of these models resulted in similar frequencies of macrophages in the spleen, BLT mice had higher frequencies in the intestinal mucosa than the Renal PedThy mice, although it was not significantly different. Leg PedThy mice had delayed detection of monocytes in the blood and had very few tissue macrophages, similar to HSC mice.

In our hands, implantation of pediatric thymus in the quadriceps of NSG mice resulted in variable and, ultimately, transient frequencies of human immune cells compared to surgical implantation under the renal capsule. In fact, while thymic tissue was detected under the renal capsule of reconstituted Renal PedThy mice, we did not grossly observe remaining thymic tissue in the Leg PedThy mice. Our results differ from the Colas et al*.* study^9^, which found similar reconstitution levels in BLT mice and mice implanted in the leg with pediatric thymus, suggesting that perhaps an insufficient amount of thymus was used in our study and that a threshold thymus amount is required for sustained reconstitution. In addition, donor variability and the method of myeloablation could influence reconstitution. Similarly, the Renal PedThy mice made in our lab were similar but had somewhat lower, more variable reconstitution compared to BLT mice obtained from the Humanized Immune System Mouse Program at the Ragon Institute, which also could reflect differences in the quantity and quality of the implanted tissue. While implantation into the quadriceps is a less invasive surgery compared to implantation under the renal capsule, introducing enough tissue into the muscle is still technically challenging.

Of the previous studies producing HIS mice from neonatal or pediatric thymus, only the study with leg implantation evaluated mucosal transmission of a recombinant lab-adapted strain of HIV-1^9^. We compared rectal transmission of HIV-1_CH185_, a T/F subtype C strain, in mice that reached the threshold of reconstitution in each group. While a group of 7 BLT mice were all infected after challenge with similar levels of plasma viremia, only 2/3 reconstituted HSC mice became infected and had significantly lower replication after the same challenge. However, when the first two Leg PedThy mice that showed peripheral hCD45+ cell reconstitution were challenged with HIV-1, neither became infected. Challenges were repeated 4 weeks later in those mice and the mice remained uninfected, which suggested that immune reconstitution was incomplete. The rest of the mice in the study were determined to be reconstituted only if hCD45+ cells were detected at ≥ 10% in peripheral blood. No other Leg PedThy mice met that criteria and, thus, were not challenged. Four of the 8 Renal PedThy mice became reconstituted and all were infected after HIV-1 challenge. However, these mice had variable viremia, suggesting different levels of virus replication compared to the BLT mice.

Work from nonhuman primates has shown that rectal transmission of simian immunodeficiency virus (SIV) results primarily in the detection of infected intraepithelial and lamina propria CD4+ lymphocytes and some myeloid cells within 1 week^23,24^. In addition, the frequency of memory CD4+ T cells expressing α_4_β_7_, the integrin that mediates homing to the intestine, is associated with susceptibility of nonhuman primates to SIV infection^25^. The virus rapidly disseminates within 1–2 weeks of challenge to the draining lymph nodes and multiple tissues^26,27^. Thus, we hypothesized that replication of HIV-1 in mice would correlate with the frequencies of both virus target cells at the site of transmission and in primary lymphoid tissues, such as the intestine and spleen, respectively. Indeed, the amount of HIV-1 replication, as measured by the plasma virus AUC after rectal challenge, correlated with the frequencies of macrophages and CD4+ T cells in the intestine as well as CD4+ T cells in the spleen. HIV-1 transmission and replication was greatest in BLT mice, followed by Renal PedThy mice, and HSC mice. The Leg PedThy mice did not become infected and had the lowest frequencies of CD4+ T cells in the intestine and the spleen.

Our results suggest that pediatric thymus can be used to reconstitute the immune system in busulfan-treated NSG mice to levels near sublethally irradiated BLT mice when implantation is performed under the renal capsule. It is possible that more tissue or donor tissues with specific genotypes may be needed to better reconstitute the mice. These mice can be infected mucosally with HIV-1 better than HSC mice. While this model avoids the use of fetal tissues, the requirement for human pediatric thymus and proficient surgical techniques may still be a barrier for some researchers to use these mice for HIV-1 studies. Improvement of transgenic HIS mice without the requirement of tissue implantation and with constant production of required human immune factors is still needed as preclinical models with functional immune cells to evaluate HIV-1 transmission, pathogenesis, therapeutics, and curative strategies prior to use in humans.

## Methods

### Animals

For PedThy and HSC mice, NSG (NOD.*Cg-Prkdc*^*scid*^*Il2rg*^*tm1Wjl*^/SzJ) mice were purchased from Jackson Laboratory and/or bred in house. BLT mice made from NSG mice were purchased from the Ragon Institute as part of a separate study and were used as a comparison for immune reconstitution and HIV-1 challenge. All animal work was conducted according to the Public Health Services. Mice were housed at the University of Pittsburgh Division of Laboratory Animal Resources in accordance with the American Association of Accreditation of Laboratory Animal Care standards and with ARRIVE guidelines. All procedures were approved by the University of Pittsburgh Institutional Animal Care and Use Committee under protocol 20026752. Animals were housed in microisolator cages and monitored daily for behavior, appearance, and physiology. Mice had unlimited water, which was autoclaved, acidified, and treated on alternate weeks with sulfamethoxazole-trimethoprim, and dry food. In addition, DietGel 76A cups (ClearH2O) often were provided in cages to provide extra nutrition post-procedure. Animals were randomized into groups. All procedures were conducted while the animals were sedated either with an intraperitoneal injection of ketamine (Henry Schein, 44 mg/kg) or inhalation of isoflurane (5% in 70% O_2_/30% NO_2_). Animals were euthanized at the endpoint of the study with 100% carbon dioxide at a flow rate of 20–30%. Any animal that failed to thrive (i.e., loss of 20% body weight from the start of the experiment), ambulate, perform normal mouse behavior, or obviously moribund was also euthanized.

CD34+ cells were isolated from cord blood or fetal liver tissues (4 donors) by bead selection (Stem Cell Technologies) and frozen viably in liquid nitrogen until use. Pediatric thymus samples (2 donors) were obtained from the University of Pittsburgh Biospecimen Core, an Institutional Review Board-approved Honest Broker System in accordance with relevant guidelines and regulations, from cardiac surgeries performed at Children's Hospital of Pittsburgh with informed consent from all subjects' legal guardians. Thymus samples were dissected into 10–15 mm^3^ pieces (approximately 600) and frozen in liquid nitrogen until use. Donor 1 was 3 years old and Donor 2 was 11 weeks old. Mice were myeloablated by sublethal irradiation (2 Gy) or 20 mg/kg busulfan at 24 and 48 h prior to surgery. A subset of animals were not myeloablated. Implantation of pediatric thymus was performed either into the quadriceps femoris muscle of one leg (Leg), after culturing for 10 days as previously described^9^, or under the renal capsule (Renal) as previously described^8^. In a subset of mice, 100 μg anti-CD2 antibody (Leinco Technologies) was injected into the mice at 0 and 7 days after tissue implantation, as previously described^8^.

Control animals consisted of mice injected with HSCs without thymus implantation and BLT mice purchased from the HIS Mouse Program.

Human immune cell reconstitution of all mice was determined by flow cytometry of whole blood drawn from mice and stained with anti-mCD45-APC (30-F11), anti-hCD45-BUV395 (HI30), anti-hCD3-PE-Cy7 (SK7), anti-hCD4-BV421 (RPA-T4), anti-hCD8α-BV650 (RPA-T8), anti-hCD14-PE (M5E2), and anti-hCD19-APC-R700 (HIB19) antibodies (BD Biosciences).

### Cells

HEK293 T were cultured in Dulbecco’s modified Eagle’s medium (Thermo Fisher Scientific) supplemented with 10% fetal bovine serum (FBS; Atlanta Biologicals), 100 U/ml penicillin, 100 μg/ml streptomycin, and 0.292 mg/ml of L-glutamine (P/S/G; Thermo Fisher Scientific). GHOST-R3/X4/R5 cells^28^ were maintained in the medium described above with the addition of 100 μg/ml of G418 (Thermo Fisher Scientific), 100 μg/ml hygromycin (Thermo Fisher Scientific), and 0.5 μg/ml puromycin (EMD Millipore).

### Rectal HIV-1 challenges

Subtype C HIV-1_CH185_ was produced from a proviral plasmid obtained from Christina Ochsenbauer by transfection of HEK293T cells with Lipofectamine 2000 (Invitrogen), as previously described^15^. HIV-1 infectivity was determined after 48 h after limiting dilution on GHOST-R3/X4/R5 cells by flow cytometry of GFP+ cells.

Mice were challenged atraumatically via the rectum with 6 × 10^4^ infectious units of HIV-1. This was determined in BLT mice as the minimum amount to infect all BLT animals intrarectally. Animals that did not have detectable viremia after 4 weeks were rechallenged in the same manner.

### Viral RNA quantitation

Plasma HIV-1 RNA was isolated as previously described^29^ and quantified by the single copy assay (SCA)^30^. Briefly, a known amount of avian sarcoma-leukosis (RCAS) virus^31^ was spiked into each 50 μl plasma sample as an internal RNA isolation control. HIV-1 and RCAS were pelleted by centrifugation at 4 °C. Total viral RNA was isolated with guanidinium isothiocyanate and glycogen and cDNA synthesis was performed with random hexamers. TaqMan qPCR was performed in duplicate for all samples and RNA standards synthesized by in vitro transcription, using the RiboMAX large scale RNA production system (Promega) or from a custom HIV-1 RNA oligo (IDT). Data were only reported from samples in which RCAS was successfully amplified. The limit of quantitation of SCA from 50 μl plasma was 120 HIV-1 RNA copies/ml plasma.

### Immunofluorescence staining

Spleens and portions of intestine were collected at necropsy from mice challenged with HIV-1 and fixed in 4% paraformaldehyde. Tissues (n = 2 per group) were embedded in OCT, sectioned, and stained as previously described^32^. The primary antibodies used were rat anti-CD3 (1:500; Abcam, clone CD3-12), rabbit polyclonal anti-CD8α (1:750; Abcam, ab4055), and mouse anti-CD68 (1:1000; Agilent, clone PG-M1). Secondary antibodies used were goat anti-rat-Cy3 (1:1000; Jackson ImmunoResearch), donkey anti-rabbit-Alexa488 (1:750; ThermoFisher), and donkey anti-mouse-Cy5 (1:1000; Jackson ImmunoResearch).

A Nikon A1 spectral inverted confocal microscope with a 40X 1.49 NS oil immersion objective (Nikon) was used to acquire images of the fixed tissue samples. LU-NV laser launch (Nikon) was used to emit lasers at 405 nm (Hoechst), 488 nm (Alexa Fluor 488), 561 nm (Cy3), and 640 nm (Cy5). For each tissue section, 5 images were acquired from different randomly chosen fields of view. NIS-Elements software (Nikon) was used to analyze fluorescence intensity and frequencies of each fluorescent signal.

### Statistics

Statistical analyses were performed by Prism 9.5.1 (GraphPad). Comparisons of cell frequencies or AUC between groups were calculated by two-tailed unpaired (peripheral blood) or paired (tissue) t tests. Correlations between different cell frequencies were calculated by two-tailed Pearson correlation.

### Supplementary Information

Supplementary Figures.

## Data Availability

The datasets used and/or analyzed during the current study will be available from the corresponding author on a reasonable request.
