# Supplementary material for: Use of pediatric thymus to humanize mice for HIV-1 mucosal transmission
Source: Sci Rep. 2023 Oct 10;13:17067. doi: 10.1038/s41598-023-44366-2 (PMC10564933; doi:10.1038/s41598-023-44366-2)

Supplementary Figure Legends

Figure S1. **Evaluation of peripheral mononuclear cells from all mice.** The gating strategy for flow cytometry of mouse peripheral blood cells is shown.

Figure S2. Comparison of thymic implants from BLT and PedThy Renal mice. (a) Photographs of representative kidneys from BLT and PedThy mice showing thymic implant sites. (b) Micrographs of thymic tissues after H&E staining are shown. Identifiable thymus is outlined in red. Arrows denote likely graft location that has been replaced by fat or connective tissue.

Figure S3. Comparison of human CD3+CD8+ and CD3+CD4+CD8+ cells in the peripheral blood of the mice. The percentages of hCD8+ cells in the T cell population are shown over time for the (a) Leg PedThy, (b) Renal PedThy, (c) HSC, and (d) BLT mice. Each symbol denotes a separate animal.

Figure S4. Immunofluorescence of hCD8+ T cells in the spleen and intestine of mice. The average frequencies of hCD3+CD8+ T cells in the spleens and intestines of HIV-challenged mice in each of the groups (2 mice/group and 5 random fields of view/tissue) are shown.

Supplementary Figure S1

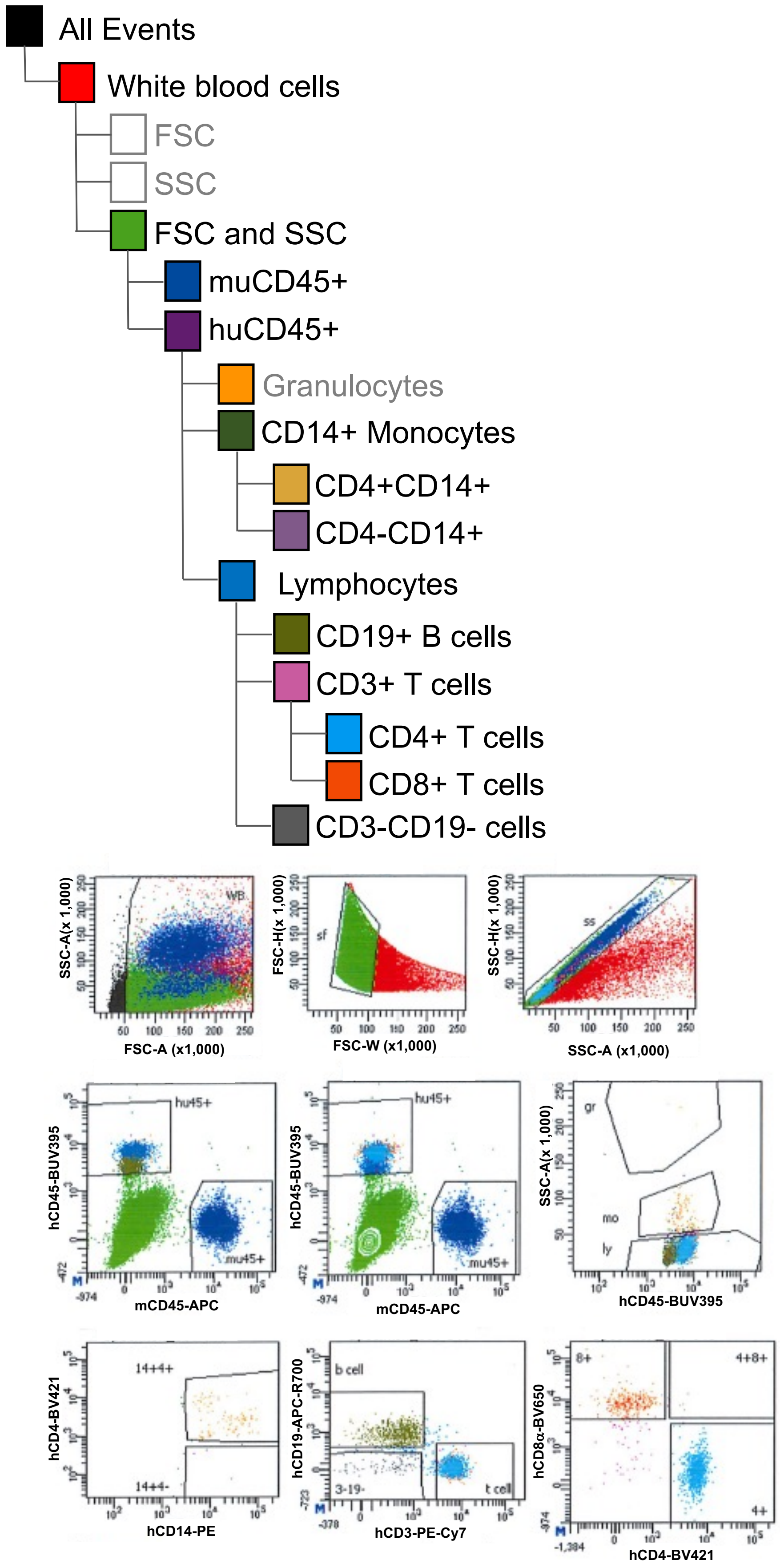

Supplementary Figure S2

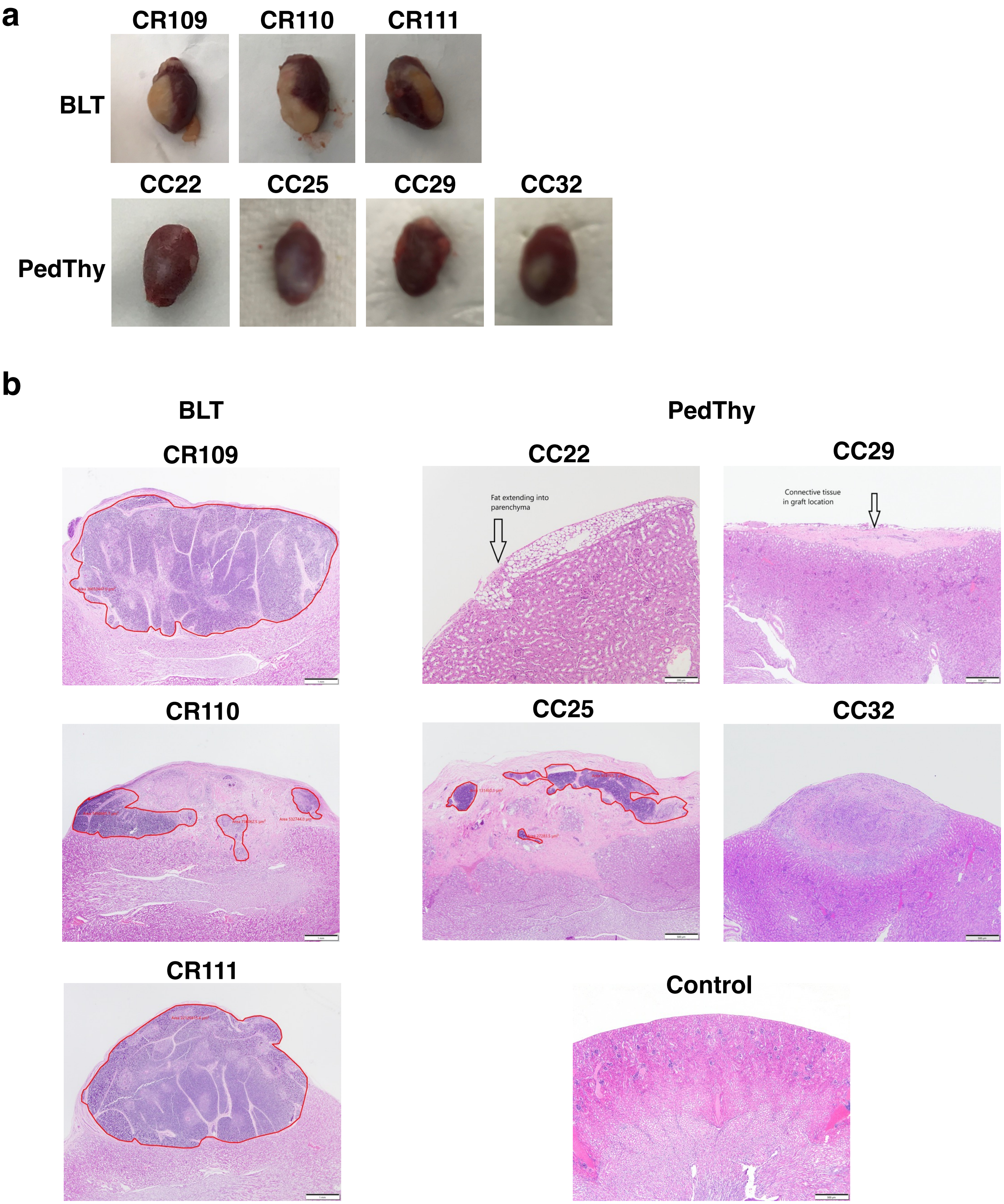

Supplementary Figure S3

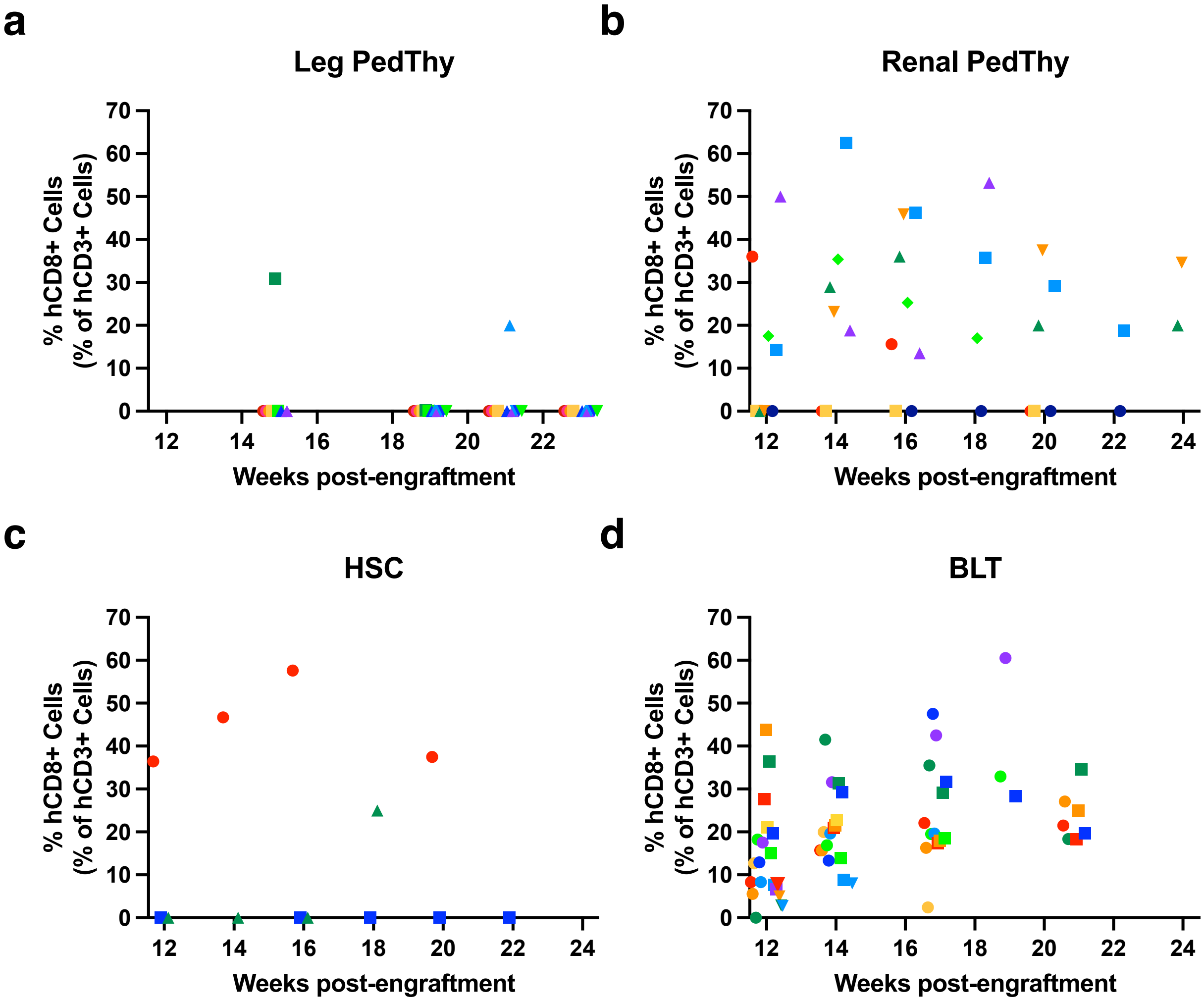

Supplementary Figure S4

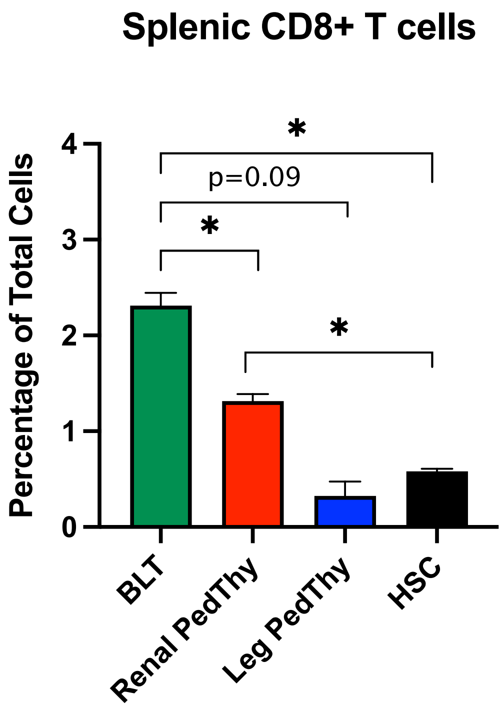

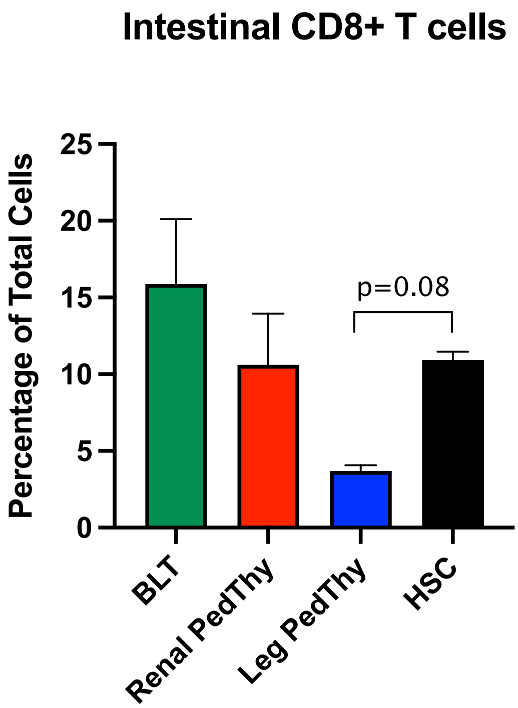

Supplement: Supplementary file 1 — Supplementary Figures. [file 41598_2023_44366_MOESM1_ESM.pdf]
